# Anti-Cancer SERCA Inhibitors Targeting Sorafenib-Resistant Human Papillary Thyroid Carcinoma

**DOI:** 10.3390/ijms24087069

**Published:** 2023-04-11

**Authors:** Hang-Seok Chang, Yonjung Kim, So Young Lee, Hyeok Jun Yun, Ho-Jin Chang, Ki Cheong Park

**Affiliations:** 1 Department of Surgery, Thyroid Cancer Center, Gangnam Severance Hospital, Institute of Refractory Thyroid Cancer, Yonsei University College of Medicine, Seoul 06273, Republic of Korea; 2 EONE-DIAGNOMICS Genome Center, New Drug R&D Center, 291 Harmony-ro, Yeonsu-gu, Incheon 22014, Republic of Korea; 3 Department of Surgery, Yonsei University College of Medicine, 50-1, Yonsei-ro, Seodaemun-gu, Seoul 03722, Republic of Korea

**Keywords:** human papillary thyroid cancer, sorafenib, anti-cancer drug-resistant papillary thyroid cancer

## Abstract

Thyroid cancer is generally curable and, in many cases, can be completely treated, although it can sometimes recur after cancer therapy. Papillary thyroid cancer (PTC) is known as one of the most general subtypes of thyroid cancer, which take up nearly 80% of whole thyroid cancer. However, PTC may develop anti-cancer drug resistance via metastasis or recurrence, making it practically incurable. In this study, we propose a clinical approach that identifies novel candidates based on target identification and validation of numerous survival-involved genes in human sorafenib-sensitive and -resistant PTC. Consequently, we recognized a sarco/endoplasmic reticulum calcium ATPase (SERCA) in human sorafenib-resistant PTC cells. Based on the present results, we detected novel SERCA inhibitor candidates 24 and 31 via virtual screening. These SERCA inhibitors showed remarkable tumor shrinkage in the sorafenib-resistant human PTC xenograft tumor model. These consequences would be clinically worthwhile for the development of a new combinatorial strategy that effectively targets incredibly refractory cancer cells, such as cancer stem cells and anti-cancer drug-resistant cells.

## 1. Introduction

Thyroid cancer (TC) is a common cancer subtype that affects the thyroid gland, which is a small gland on the base of the neck. TC is reported most commonly in the 30 s and in those aged above 60 years. More than 90% of all endocrine cancers are widely known as TC, and the incidence ratio has grown over the past four decades, making it the most common endocrine malignancy [1]. TC is conventionally classified into four categories [2,3,4]. In addition, TC is generally categorized into differentiated and undifferentiated classes in proportion to clinical exposure [5,6]. Well-differentiated TC (WDTC) conventionally showed a suitable response to therapy, whereas undifferentiated TC (UTC) and poorly differentiated TC (PDTC) are infrequent and aggressive, metastatic, and have a poor prognosis [7,8]. Despite PTC being a WDTC, anti-cancer drug-resistant TC is a fundamentally critical topic that causes patient death via recurrence or metastasis [9,10]. It is well known that clinical behavior based on molecular and biological mechanisms is different between anti-cancer drug-sensitive cancer and resistant cancer [11,12,13]. Therefore, several studies have shown that the range of mutations affects anti-cancer drug-sensitive and -resistant cancers [14,15,16,17,18,19]. Many previous studies have demonstrated molecular diversities to clarify the poor prognosis of PTC in anti-cancer drug-resistant patients; however, to the best of our knowledge, no study has yet provided a clear description of the underlying mechanism. Refractory PTC constantly acquires drug resistance; therefore, there is a need for effective clinical approaches [20,21,22]. SERCA is a critical controller of cytosolic-free calcium [23,24,25]. The management of cytoplasmic-free calcium is influenced by many processes, including cellular death, autophagy, and apoptosis under acute endoplasmic reticulum (ER) stress [12,25,26]. In addition, we identified novel SERCA inhibitor compounds 24 and 31, which may be potential therapeutic agents for sorafenib-resistant PTC.

Our findings could be clinically important for the development of innovative target-mediated combinatorial plans that efficaciously and selectively target highly malignant cancer cells, such as sorafenib-resistant cancer cells.

## 2. Results

### 2.1. Characteristics of Human PTC Cell Lines

The present study employed four types of PTC cell lines obtained from patient specimens (Table 1): YUMC-S-P1 (sorafenib-sensitive PTC cell line) and YUMC-R-P1, -P2 [27], and -P3 [27] (sorafenib-resistant PTC cell lines, respectively) were isolated from patients specimen under PTC care at the Severance Hospital, Yonsei University College of Medicine, Seoul, Republic of Korea. Sorafenib-resistant PTC cases were frequently observed with metastasis or recurrence as well as more aggressive than sorafenib-sensitive PTC (Table 1).

### 2.2. Distinction of Genetic and Signaling Pathways between Human Sorafenib-Sensitive and -Resistant PTC Cell Lines

**Figure 1:**
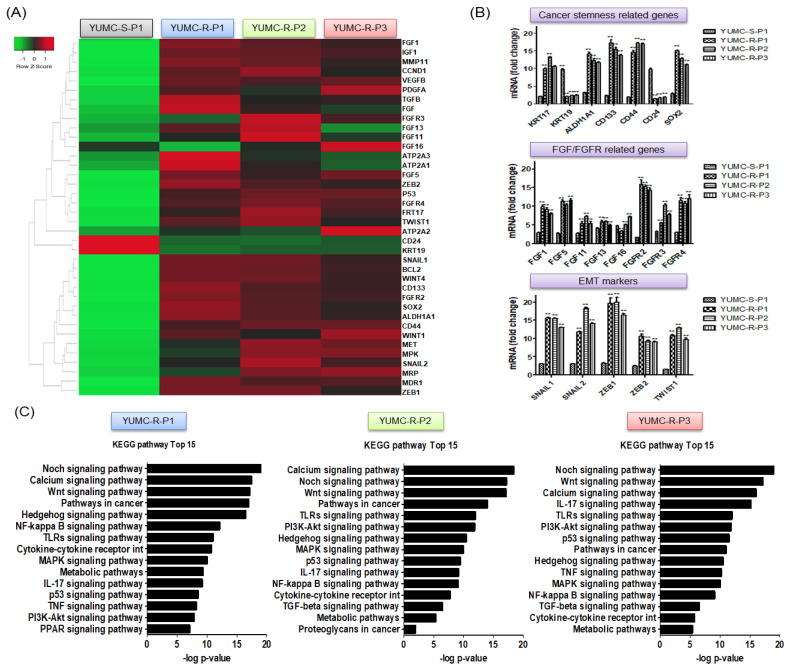

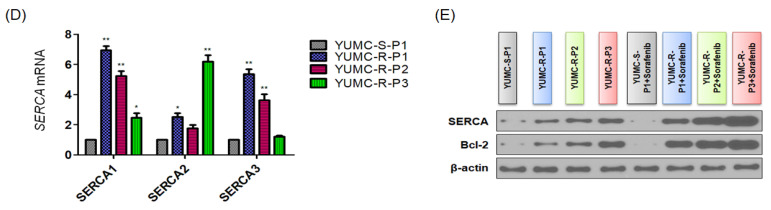
Features of all investigated papillary thyroid cancer (PTC) cell lines. (**A**) Hierarchical clustering of genes indicating divergent gene expression and gene expression profile changes between human sorafeni-sensitive and -resistant PTC cells. (**B**) Analysis of gene expression level based on mRNA-Seq for epithelial–mesenchymal transition (EMT), fibroblast growth factor (FGF)/FGF receptor, and cancer stem cell (CSC) markers in sorafenib-sensitive and -resistant PTC cells. (**C**) Bar plot revealing remarkably increased expression of 15 signaling pathways in soraenib-resistant PTC cells, YUMC-R-P1 (**Left**), YUMC-R-P2 (**middle**), and YUMC-R-P3 (**Right**). (**D**,**E**) Expression levels of SERCA RNA and protein in sorafenib-sensitive and -resistant PTC cells in the absence and presence of sorafenib treatment. * *p* < 0.05 vs. sorafenib-sensitive PTC cells (YUMC-S-P1), ** *p* < 0.01 vs. sorafenib-sensitive PTC cells (YUMC-S-P1).

To assess the signaling pathways and genetic distinctions between sorafenib-sensitive (YUMC-S-P1) and sorafenib-resistant (YUMC-R-P1, -P2, and -P3) PTC, we accomplished RNA sequencing (Figure 1A–C). Results showed that entirely differently expressed genes showed that YUMC-R-P1, -P2, and -P3 cells were involved in the decisive growth of the *EMT* (epithelial-mesenchymal transition) markers *ZEB* (zinc finger E-box-binding homeobox), *TWIST* (twist family bHLH transcription factor), and *SNAIL* (zinc finger protein SNAI1), and they were contrasted to YUMC-S-P1 (Figure 1A). EMT- and FGF/FGFR (fibroblast growth factor/Fibroblast growth factor receptors)-related genes with the most marked growth were *SNAIL*, *ZEB*, *TWIST*, *FGF*, and *FGFR* (Figure 1B, top and middle) in sorafenib-resistant PTC. Cancer stem cell (CSC)-, EMT-, and FGF-associated genes were greatly expressed in sorafenib-resistant PTC compared to sorafenib-sensitive PTC (Figure 1B, bottom). Kyoto Encyclopedia of Genes and Genomes (KEGG) pathway analysis revealed that cancer stemness-related signaling pathways (Wnt, Hedgehog, PPAR, calcium, PI3K/Akt, and TGF/SMAD signaling pathways) [28] were markedly elevated in sorafenib-resistant PTC compared with that in sorafenib-sensitive PTC (Figure 1C, left, middle, and right). We focused on the signaling pathway of calcium that was enormously induced in sorafenib-resistant PTC cells. Furthermore, there was a slight difference between *SERCA* isoforms; however, of particular importance was the basal expression level of *SERCA*, which is an acute regulator of calcium homeostasis and anti-apoptosis [24,29,30,31]. These were increased in YUMC-R-P1, -P2, and -P3 sorafenib-resistant PTC cells compared with that in sorafenib-sensitive PTC cells, YUMC-S-P1 (Figure 1D). In addition, when sorafenib was administered, SERCA protein expression in sorafenib-resistant PTC was significantly higher than that in sorafenib-sensitive PTC (Figure 1E).

Taken together, these results suggest that sorafenib-resistant PTC may be of significant value for therapeutic access and management of metastasis or recurrence in patients with aggressive PTC. These results show that the enhancement of gene pathways in the regulation of SERCA and cancer stemness is important for prolonging the survival of sorafenib-resistant PTC cells under conditions of sorafenib treatment.

### 2.3. Target-Specific Observation of the Novel Therapeutic Approach of Candidates 24 and 31, SERCA Inhibitors That Suppressed the Survival of Sorafenib-Resistant PTC Cells, for Curing Human Sorafenib-Resistant PTC by Using in Silico Screening

**Figure 2:**
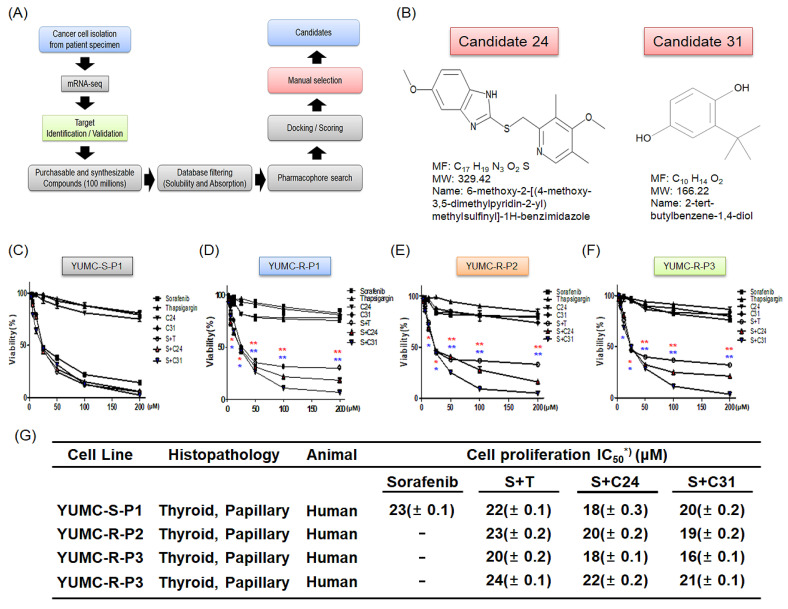

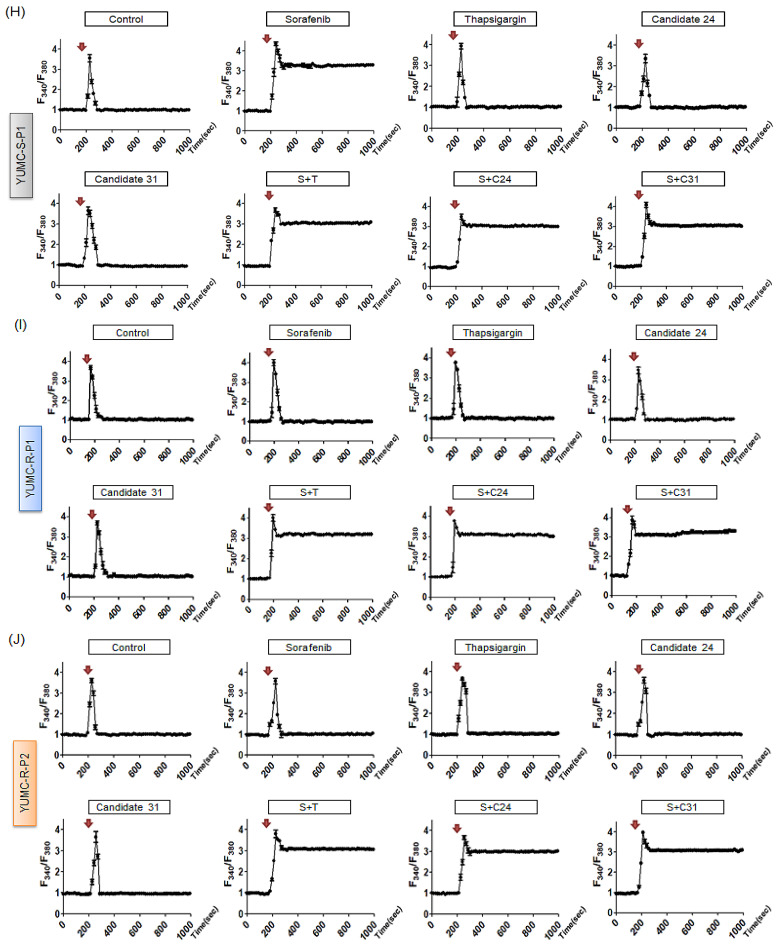

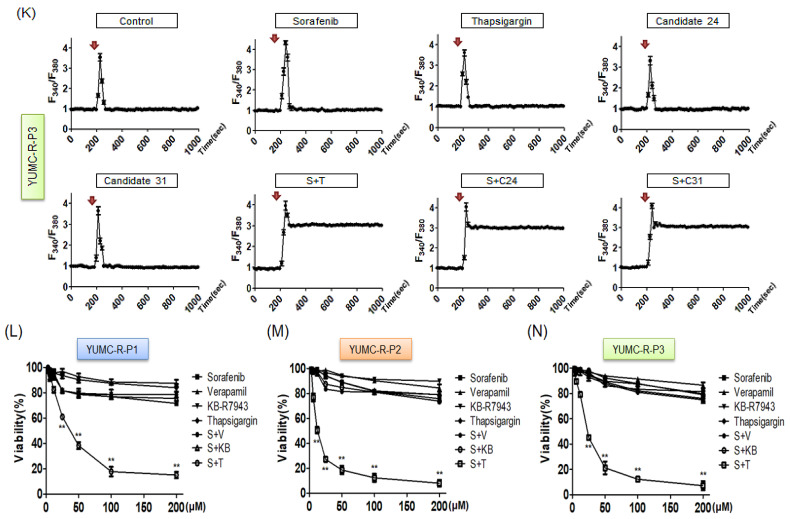
Plot of the study plan and in silico screening using the ECBS method to determine novel SERCA inhibitors. (**A**) The schematic diagram for determining novel SERCA inhibitors using the ECBS method. (**B**) Information and chemical structures of the novel SERCA inhibitors, candidates 24 and 31. (**C**–**F**) Combinational anti-cancer effectiveness of sorafenib and novel SERCA inhibitors on sorafenib-sensitive and -resistant PTC cells. Cell viability of sorafenib-sensitive ((**C**), YUMC-S-P1) and sorafenib-resistant ((**D**); YUMC-R-P1, (**E**); -R-P2, (**F**);-R-P3) PTC cells treated with sorafenib alone or combined with SERCA inhibitors. Points express the mean percentage of the values shown in the solvent-treated control. All experiments were repeated at least thrice. Data represent the mean ± standard deviation. * *p* < 0.05 and ** *p* < 0.01 versus control. *; C24 and *; C31. (**G**) IC_50_ values for combinational treatment with SERCA inhibitors and sorafenib in sorafenib-sensitive and -resistant PTC cells. Each datum shows the mean of triplicate. SEM, standard error of the mean; MTT, 3-(4,5-dimethylthiazol-2-yl)-2,5-diphenyltetrazolium bromide; IC_50_, half-maximal inhibitory concentration. The asterisk describes the lowest half-maximal inhibitory concentration. (**H**–**K**) Cytosolic-free calcium was measured after treatment with sorafenib, thapsigargin (SERCA inhibitor, positive control), and novel candidates (24 and 31) in sorafenib-sensitive PTC and -resistant PTC. An arrow indicates the addition of high K^+^ depolarization to increase growth in Ca^2+^ on traces. (**L**–**N**) Cell viability assay of treatment with calcium channel blocker (verapamil), Na^+^/Ca^2+^ exchanger (NCX) inhibitor (KB-R7943), and SERCA inhibitor (thapsigargin) alone or combined with sorafenib. * *p* < 0.05 and ** *p* < 0.01 versus control.

We formulated a hypothesis based on Figure 1 that in sorafenib-resistant PTC cells, the restriction of SERCA would be a practicable clinical overture for sorafenib resistance. We identified SERCA binding compounds and the probability of pharmacophoric binding modes via in silico screening. Consequently, 1350 (prerogative on docking score), 124 (manual selection), and 11 candidates (natural product included) compounds were identified (Figure 2A). Of these, two candidates, 24 and 31, showed a high binding affinity to SERCA, indicating critical inhibition of SERCA, and these two candidates were eventually selected as SERCA inhibitors (Figure 2B). We used an ECBS (evolutionary chemical binding similarity) for the identification of candidates 24 and 31 as potential novel SERCA inhibitors for the treatment of sorafenib-resistant PTC. Moreover, to assess the anti-cancer influence of candidates 24 and 31 and their association with sorafenib, we performed cell viability assays for sorafenib-sensitive (YUMC-S-P1) and -resistant (YUMC-R-P1, -P2, and -P3) PTC cells administrated with sorafenib alone or with candidate 24 or 31 (Figure 2C–F). The viability of YUMC-S-P1 cells markedly diminished in a dose-reliant method following sorafenib administrate with or without candidate 24 or 31 (Figure 2C). However, the viabilities of YUMC-R-P1, -P2, and -P3 cells showed no notable difference following sorafenib administration. Specifically, thapsigargin (positive control, established SERCA inhibitor) or candidate 24 or 31 (novel SERCA inhibitor candidates) combined with sorafenib administration noticeably restricted the viability of sorafenib-resistant PTC cells in a dose-reliant method (Figure 2D–F). The administration of thapsigargin or candidate 24 or 31 alone did not considerably affect the viability of sorafenib-sensitive and -resistant PTC cells. The half-maximal inhibitory concentration (IC_50_) of sorafenib was 23 µM in sorafenib-sensitive PTC (Figure 2G). There was no significant difference in the IC_50_ of sorafenib treatment alone compared to its combinatorial administration with thapsigargin or novel candidates. In sorafenib-resistant PTC, the anti-cancer efficacy of sorafenib treatment alone was extremely weak (Figure 2G). Nonetheless, the anti-cancer efficacy of sorafenib was exceedingly strong when combined with SERCA inhibitors (thapsigargin or candidate 24 or 31) (Figure 2G). In combination with thapsigargin or candidates 24 and 31, the IC_50_ of sorafenib was approximately 16–24 μM in YUMC-R-P1, -P2, and -P3 (Figure 2G).

We measured the variation of intracellular Ca^2+^ levels with microspectrofluorimetry. Cytosolic Ca^2+^ was measured in relation to high K^+^ depolarization to increase growth in cytosolic Ca^2+^. Furthermore, cytosolic Ca^2+^ clearance was estimated under treatment with sorafenib and SERCA inhibitor (thapsigargin, candidate 24 and 31) either alone or combined with sorafenib-sensitive and -resistant PTC cells (Figure 2H–K). The cytosolic Ca^2+^ levels in sorafenib-sensitive PTC cells failed to return to the basal levels in the presence of sorafenib regardless of the combination with SERCA inhibitors (Figure 2H). In sorafenib-resistant PTC cells, there were no critical changes in the levels of cytosolic Ca^2+^ treatment with sorafenib or SERCA inhibitor alone (Figure 2I–K). Moreover, the combined treatment with sorafenib and SERCA inhibitor failed to return to the basal levels after the spike of intracellular Ca^2+^ (Figure 2I–K). These differences in cytosolic Ca^2+^ levels between sorafenib-sensitive and -resistant PTC cells might be correlated with SERCA expression levels (Figure 1E). To confirm that the main regulator for prolonged survival after treatment with sorafenib in sorafenib-resistant PTC cells was SERCA and not calcium ion channels and NCX, we conducted a cell viability assay treated with calcium channel blocker (verapamil), Na^+^/Ca^2+^ exchanger (NCX) inhibitor (KB-R7943) and SERCA inhibitor (thapsigargin) alone or combined with sorafenib (Figure 2L–N). Calcium channel blocker, NCX, and SERCA inhibitors treated alone showed no critical changes, while that of treatment with SERCA inhibitor failed to survive in a dose-dependent manner under treatment with sorafenib (Figure 2L–N).

These results showed that SERCA might play an indispensable role in prolonging survival in patients with sorafenib-resistant cancer when administered with chemotherapy via regulating the overloaded cytosolic-free calcium.

### 2.4. Combinatorial Treatment of Sorafenib with SERCA Inhibitors Induces ER Stress-Mediated Apoptosis in Sorafenib-Resistant PTC Cells

**Figure 3:**
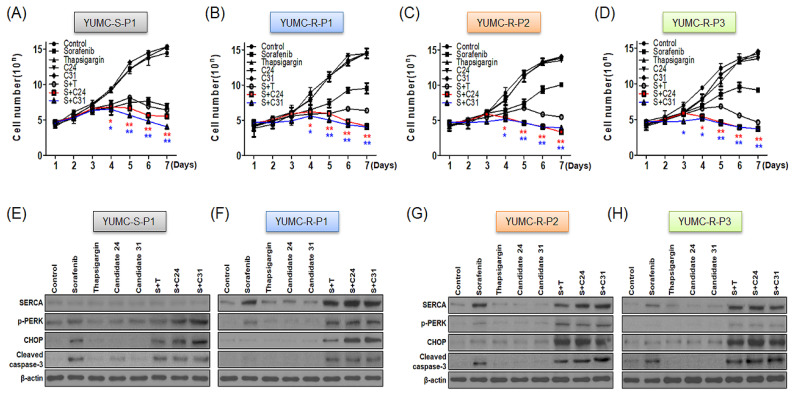
Inhibition of cell proliferation and apoptosis level estimated using cell proliferation and immunoblot analysis. Cell proliferation (**A**–**D**) and immunoblot analysis (**E**–**H**) of human sorafenib-sensitive (**A**,**E**), YUMC-S-P1) and -resistant ((**B**,**F**), YUMC-R-P1; (**C**,**G**), YUMC-R-P2; (**D**,**H**), YUMC-R-P3) PTC cells. (**A**–**D**) Human PTC cells were presented with sorafenib and SERCA inhibitors, either combined or alone. (**E**–**H**) Immunoblot analysis with SERCA, p-PERK, and CHOP (endoplasmic reticulum stress marker), and cleaved-caspase-3 (apoptosis-related marker) in sorafenib-sensitive (YUMC-S-P1) and -resistant (YUMC-R-P1, -P2, and -P3) PTC cells. * *p* < 0.05 and ** *p* < 0.01 versus control. *; C24 and *; C31.

To prove the combinatorial anti-cancer efficacy of sorafenib and novel SERCA inhibitors (candidates 24 and 31), we evaluated cell proliferation (Figure 3A–D) and executed an immunoblot assay to assess the expression of ER stress markers in sorafenib-sensitive and -resistant PTC cells treated with each agent alone or in combination with sorafenib and SERCA inhibitors (thapsigargin and candidate 24 or 31) (Figure 3E–H). In sorafenib-sensitive PTC, sorafenib significantly restricted cell proliferation regardless of its administration alone or in combination with SERCA inhibitors (Figure 3A). Treatment with SERCA inhibitor alone did not influence cell proliferation. Cell proliferation in sorafenib-resistant PTC was markedly suppressed by the combination of sorafenib and SERCA inhibitors (Figure 3B–D). Sorafenib or SERCA inhibitors alone did not show any significant effect in sorafenib-resistant PTC (Figure 3B–D). SERCA protein expression did not change significantly in sorafenib-sensitive PTC, regardless of whether the cells were treated with sorafenib alone (after 24 h) or in combination (after 24 h) with SERCA (Figure 3E). Consequently, sorafenib, regardless of treatment alone or in combination with SERCA inhibitors, showed high induction of ER stress-mediated apoptosis through the induction of phosphorylated PERK and CHOP (markers of ER stress) as well as cleaved-caspase 3 (apoptotic marker) (Figure 3E). Severe ER stress-mediated apoptotic activity could be avoided in sorafenib-resistant PTC by induction of SERCA protein under severe ER stress conditions, such as with sorafenib treatment (Figure 3F–H). However, combinatorial treatment with sorafenib and SERCA inhibitors significantly induced the expression of ER stress (p-PERK and CHOP) and apoptotic (cleaved-caspase 3) markers via functional inhibition of SERCA in drug-resistant PTC (Figure 3F–H).

Collectively, these results indicate that the novel SERCA inhibitors, candidates 24 and 31, could be potent agents against sorafenib-resistant PTC.

### 2.5. Novel Therapeutic Approaches of Candidates 24 and 31 in Xenograft Mouse Model of Human Sorafenib-Resistant PTC

**Figure 4:**
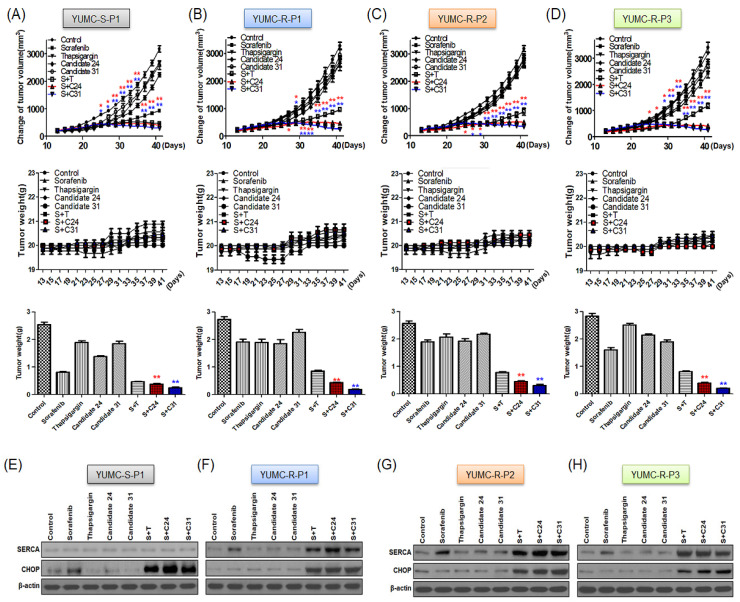
Combined therapy of sorafenib and novel SERCA inhibitors increased tumor shrinkage more effectively in human sorafenib-resistant PTC cells (YUMC-R-P1, -P2, and -P3) than in a human sorafenib-sensitive (YUMC-S-P1) xenograft model. ((**A**–**D**), **top**) Changes in tumor volume, ((**A**–**D**), middle) Changes in whole-body weight, and ((**A**–**D**), **bottom**) the resected tumor weight (each group, *n* = 10). Tumor size was estimated in NOD/Shi-scid IL-2Rγ KOJic (NOG) mice, where animals were presented with each agent alone or with either sorafenib joined with novel SERCA inhibitors (candidates 24 or 31). (**E**–**H**) Immunoblot analysis with SERCA and CHOP (endoplasmic reticulum stress marker) in a mouse xenograft model with sorafenib-sensitive (YUMC-S-P1) and -resistant (YUMC-R-P1, -P2, and -P3) PTC cells. Data symbolize the mean ± standard error of the mean. * *p* < 0.05 and ** *p* < 0.01, compared to the control. *; C24 and *; C31.

To evaluate the combinatorial anti-cancer efficacy of novel SERCA inhibitor candidates 24 and 31, we used a mouse xenograft model with sorafenib-sensitive (YUMC-S-P1) and -resistant PTC (YUMC-R-P1, -P2, and -P3) cells administrated with sorafenib alone or with SERCA inhibitors (thapsigargin or candidates 24 and 31). The detailed protocol can be found in Supplementary Materials (Figure S1). Sorafenib was used to induce genotoxic stress in the mouse xenograft model. In the xenograft model with sorafenib-sensitive PTC cells, tumor shrinkage was markedly induced by sorafenib treatment with or without the SERCA inhibitors (Figure 4A, top). In the sorafenib-resistant PTC xenograft model, sorafenib treatment alone did not significantly induce tumor shrinkage, whereas combinatorial treatment with sorafenib and candidate 24 or 31 showed markedly increased tumor shrinkage (Figure 4B–D, top). The resected tumor weight was similar to the change in tumor volume, and the resected tumor weight of sorafenib-sensitive PTC remarkably decreased after treatment with sorafenib (Figure 4A, bottom). There was no significant difference between sorafenib treatment with or without SERCA inhibitors (Figure 4A, bottom). The tumor weight in sorafenib-resistant PTC was not significantly influenced by sorafenib, whereas combinatorial treatment with sorafenib and candidate 24 or 31 induced a marked decrease in tumor weight (Figure 4B–D, bottom). Treatment with SERCA inhibitors (thapsigargin or candidate 24/31) alone did not noticeably influence the whole-body weight of mice (Figure 4A–D, middle). In the immunoblot assay of tumor tissue, SERCA expression did not significantly change with sorafenib treatment alone or in combination with SERCA inhibitors in sorafenib-sensitive PTC (Figure 4E). Sorafenib significantly induced CHOP, regardless of combination with SERCA inhibitors (Figure 4E). In contrast to sorafenib-sensitive PTC, sorafenib-resistant PTC showed high SERCA expression following treatment with sorafenib alone or in combination with SERCA inhibitors (Figure 4F–H). Meanwhile, functional inhibition of SERCA by the novel SERCA inhibitors, candidates 24 and 31, led to severe ER stress (induction of CHOP) (Figure 4F–H).

These results proved that candidates 24 and 31 may be novel therapeutic approaches for marked tumor shrinkage in a xenograft tumor model of human sorafenib-resistant PTC.

## 3. Discussion

PTC is commonplace endocrinological cancer that has a good prognosis [32,33]. However, a rare occurrence of anti-cancer drug-resistant PTC shows a poor prognosis via recurrence or metastasis, and these consequences can be associated with patient death. Various studies have shown that recurrent or metastatic PTC is refractory to most medical treatments [8,34,35,36,37]. These refractory PTCs usually have a slow; however, following anaplasia of the injury, they are altered to poorly differentiated and undifferentiated cancer, described by sharp expansion involving a poor prognosis [38,39]. In tumorigenesis of refractory TC, various cytogenetic events, as well as oncogenic mechanisms, occur [40,41,42,43]. The stemness and aggressiveness of refractory PTC have not yet been revealed. Outcomes from the advancement of anti-cancer drugs finding many studies have described that pre-operative chemotherapy can extend the survival rates after surgery [44,45,46,47]. Nonetheless, no selective therapeutic options have been accepted as the basal adjuvant or neoadjuvant background for drug-resistant cancer [48,49,50], and a substantial number of anti-cancer drug-resistant cancer patients have died; therefore, unmet medical needs have consistently increased. Refractory cancer due to drug resistance, which mediates metastasis and recurrence, is a decisive part of unmet medical needs [51,52,53,54]. Anti-cancer drug resistance remains a fundamental challenge in the treatment of patients with refractory cancer [55,56,57,58]. For these reasons, anti-cancer drug resistance in refractory cancer represents a primary challenge in cancer therapy [56,59,60].

In the present study, we conducted mRNA-Seq analysis based on ECBS with human sorafenib-resistant PTC cells to establish a therapeutic plan for refractory cancer. We performed KEGG pathway analysis, which showed that hedgehog and calcium signaling pathways were part of the 15 significantly induced signaling pathways in human sorafenib-resistant PTC compared to those in human sorafenib-sensitive PTC. Additionally, mRNA-Seq analysis exhibited that SERCA expression was dominant in sorafenib-resistant PTC cells. Our results, in light of the divergent origin of cell cultures, showed that regardless of PTC or PDTC, target genes were similarly induced in sorafenib-resistant TC. We noticed and targeted SERCA, a calcium-mediated apoptosis regulator among so many highly induced target genes. The relationship between the Notch and calcium signaling pathway has been well researched in previous studies [61,62,63], and the Notch signaling pathway is regulated by SERCA suppression [64,65]. Accordingly, we mainly focused on the decisive genes and signaling pathways involved in calcium homeostasis and survival in sorafenib-resistant PTC cells. All findings or research on anti-cancer drugs ultimately focus on killing only cancer cells. In this study, despite the broadly known anti-cancer efficacy of sorafenib, it did not notably influence human sorafenib-resistant PTC cells. In our published papers, we proposed that metabolic stress-resistant cancer cells evaded apoptosis by cytoplasmic-free Ca^2+^ overburden via SERCA increase under severe ER stress conditions [31,66,67]. The present study showed that regardless of the extreme increase in SERCA expression in sorafenib-resistant PTC cells, the functional restriction of SERCA by the novel SERCA inhibitors, candidates 24 or 31, could lead to severe ER stress-mediated apoptosis. Candidates 24 and 31 showed a significant increase in sorafenib-resistant PTC cell death due to the functional inhibition of SERCA under severe ER stress conditions. The findings of the present study may be valuable for establishing prospective logical clinical approaches for patients with refractory PTC for the progress of effective therapy. Further studies are needed to evaluate this therapeutic approach. In addition, further studies are needed because of the constraints on several patient results in this study.

Nevertheless, the present research proposes that the clinical approach based on genetic changes between sorafenib-sensitive and -resistant PTC is viable for the management of patients with refractory PTC and anti-cancer drug-resistant properties.

## 4. Materials and Methods

### 4.1. Ethical Considerations and Study Design

This study was a retrospective, single-center analysis of patients who received a diagnosis of PTC (between February 2007 and October 2020), as detailed in our previous study and Supplementary Methods.

### 4.2. Patients

A total of four types of human PTC were isolated from resected human specimens. Patient characteristics and clinical features were described as Supplementary Methods.

### 4.3. Patient Tissue Specimens for Primary Culture

Fresh tumors were resected from patients with histologically and biochemically confirmed PTC by pathological reading. These patients were cured at Severance Hospital, Yonsei University College of Medicine (Seoul, Republic of Korea). Fresh tumors were collected throughout the surgical excision of the metastatic and primary sites of PTC.

### 4.4. Tumor Cell Isolation and Primary Culture

For the elimination of fat or normal tissues, tissues were rinsed several times using 1X Hank’s Balanced Salt Solution. Tumor specimens were minced in Eppendorf or cryo tube with dissociation medium containing RPMI1640 (Roswell Park Memorial Institute-1640) and 20% fetal bovine serum (FBS) supplemented with 1 mg/mL collagenase type IV (Sigma-Aldrich, St. Louis, MO, USA; C5138). The detailed protocol can be found in our previous study [68].

### 4.5. mRNA-Seq Data

We preprocessed the raw reads from the sequencer to remove low-quality and adapter sequences before analysis and aligned the processed reads to Homo sapiens (GRCh37) using HISAT v2.1.0. The detailed protocol can be found in our previous study [12] and Supplementary Methods.

### 4.6. Whole RNA Extraction and Quantitative Real-Time Reverse Transcription RT-PCR (qRT-PCR)

Total RNA was arranged from patient-derived TC cells by extraction with the RNeasy Mini Kit (Qiagen, Valencia, CA, USA; 74106) and the One-Step RT-PCR Kit (Qiagen; 204243) following the manufacturer’s protocols. All data were normalized to the expression level of α tubulin. Primers for SERCA1, SERCA2, and SERCA3 are listed in Supplementary Table S1.

### 4.7. Statistical Analysis of Gene Expression Level

The relative abundances of genes were measured in read counts using StringTie. Further details of the protocol can be found at ‘www.frontiersin.org’ (accessed on 11 October 2022). The detailed protocol can be found in our previous study [27] and Supplementary Methods.

### 4.8. Hierarchical Clustering

Hierarchical clustering analysis was performed using complete linkage and Euclidean distance as a measure of similarity to display the expression patterns of differentially expressed transcripts that satisfied |fold change| ≥ 2 and independent *t*-test (raw *p* < 0.05). All data analysis and visualization of differentially expressed genes were conducted using R 3.5.1 (www.r-project.org, accessed on 21 August 2022). The detailed protocol can be found in our previous study [27].

### 4.9. Cell Culture

The human PTC cell lines YUMC-S-P1, YUMC-R-P1, -P2, and -P3 were obtained by tumor cell isolation from the patients and cultured in RPMI-1640 medium with 15% FBS (authenticated by using short tandem repeat profiling/karyotyping/isoenzyme analysis).

### 4.10. Cell Viability Assay

Cell viability assay was performed with the 3-(4,5-Dimethylthiazol-2-yl)-2,5-diphenyltetrazolium bromide (MTT). The cells were cultured in 96-well plates and incubated overnight to achieve 80% confluency. Cell viability of human PTC cell lines was measured after various dose-dependent manner treatments with sorafenib and SERCA inhibitors (thapsigargin and candidate 24 or 31), either using each agent alone or in combination (excluding the combination of SERCA inhibitors). The detailed protocol can be found in our previous study [27]. Data are expressed as the percentage of the signal observed in vehicle-treated cells and are shown as the mean ± SEM of triplicate experiments.

### 4.11. Cell Proliferation Assay

To assess proliferative activity, human cells were seeded into RPMI-1640 medium with sorafenib and SERCA inhibitors, each agent alone or in combination. Each agent (IC_50_)-containing medium was changed every 24 h for a total of seven days. Cell number counting is performed using trypan blue staining, followed by microscopic quantification using a hemacytometer. This experiment was repeated in triplicate, and the results were averaged.

### 4.12. Immunoblot Analysis

Primary antibodies for SERCA (1:200, #271669; Santa Cruz Biotechnology, CA, USA), C/-EBP homologous protein (CHOP, 1:100, Santa Cruz Biotechnology, #7351), Bcl-2 (1:500, # 4223S; Cell Signaling Technology, Danvers, MA, USA), p-PERK (1:500, #156919; Abcam, Cambridge, U.K.), caspase-3 (1:200, #56053; Santa Cruz Biotechnology), and β-actin (1:2000, #47778; Santa Cruz Biotechnology) were purchased and maintained overnight at 4 °C. The detailed protocol can be found in our previous study [27].

### 4.13. Intracellular Calcium Measurements by Microspectrofluorimetry

The intracellular Ca^2+^ level of sorafenib-sensitive and -resistant PTC cells were represented with a calcium-sensitive fluorescent indicator, Fura-2AM. Further protocol details are described in Supplementary Methods and published previously [12,68].

### 4.14. Human PTC Cell Xenograft

YUMC-S-P1, YUMC-R-P1, -P2, and -P3 human PTC cells (4.0 × 10^6^ cells/mouse) were cultured in vitro and injected subcutaneously into the upper left flank region of female NOD/Shi-scid IL-2Rγ KOJic (NOG) mice. Detail protocol was described in Supplementary Methods.

## 5. Conclusions

SERCA activation, known as induced cytoplasmic Ca^2+^ influx into the ER, inhibited cytoplasmic Ca^2+^ overburden and was essentially responsible for cellular resistance to apoptosis and genotoxic stress triggered by sorafenib administration. We vote therapeutic access for a noticeable increase in tumor shrinkage using a xenograft model of human sorafenib-resistant PTC cells using candidates 24 and 31, which are novel SERCA inhibitors. Clinically, these results have meaningful implications for the development of novel combinatorial therapies that target the selective vulnerability of extremely malignant cells, such as anti-cancer drug-resistant cancer cells.

## Figures and Tables

**Table 1:** Patient characteristics, clinical features, and information on human sorafenib-sensitive and -resistant papillary thyroid cancer (PTC). YUMC-R-P2 (PDTC) and -P3 were investigated in our previous article [27].

	YUMC-S-P1	YUMC-R-P1	YUMC-R-P2	YUMC-R-P3
**Age at Diagnosis**	53	54	57	34
**Sex**	Male	Female	Male	Female
**Primary Disease Site**	Thyroid	Thyroid	Thyroid	Thyroid
**Stage**	T4aN1bM0	T4aN1bM1	T3N1bM1	T4aN1bM1
**Primary Pathology**	Papillary thyroid cancer	Papillary thyroid cancer (recurrence and metastasis after sorafenib treatment)	Poorly differentiated thyroid cancer (recurrence and metastasis after sorafenib treatment)	Papillary thyroid cancer (recurrence and metastasis after sorafenib treatment)
**Classification Of Specimen Used For Culture**	Fresh tumor	Fresh tumor	Fresh tumor	Fresh tumor
**Obtained From**	Severance Hospital, Seoul, Korea	Severance Hospital, Seoul, Korea	Severance Hospital, Seoul, Korea	Severance Hospital, Seoul, Korea

## Data Availability

The data presented in this study are available upon reasonable request from the corresponding author.

## Supplementary Materials

The following supporting information can be downloaded at: https://www.mdpi.com/article/10.3390/ijms24087069/s1.
