# Supplementary material for: Anti-Cancer SERCA Inhibitors Targeting Sorafenib-Resistant Human Papillary Thyroid Carcinoma
Source: Int J Mol Sci. 2023 Apr 11;24(8):7069. doi: 10.3390/ijms24087069 (PMC10138651; doi:10.3390/ijms24087069)

Supplementary Methods

1.1. Ethical considerations and study design

This study was a retrospective, single-center analysis of patients who received a diagnosis of PTC (between February 2007 and October 2020). All procedures involving patients were performed in accordance with the institutional ethical standards, applicable local/national regulations, and guidelines of the 1964 Helsinki Declaration and its later amendments. In accordance with the Bioethics and Safety Act of Korea, formal written consent was not required for this retrospective observational analysis. The study protocol was approved by the Institutional Review Board (IRB) of Severance Hospital, Yonsei University College of Medicine (IRB protocol: 3-2019-0281). Cell samples were obtained from the patients at Severance Hospital, Yonsei University College of Medicine, Seoul, Korea.

1.2. Patient characteristics

1.2.1. Patient 1

YUMC-S-P1 was a 53-year-old man with papillary thyroid cancer (PTC). The patient had bilateral thyroid tumors with extrathyroidal extension. The patient underwent bilateral total thyroidectomy and bilateral modified radical neck dissection with central compartment neck dissection. Surgical findings showed that the tumor had invaded the recurrent laryngeal nerve, which was removed by careful shaving. After surgery, he was administered high-dose radioiodine ablation therapy three times. Recent radiologic examinations and thyroid hormone tests showed no recurrence. The pathology report indicated the presence of PTC with bilateral metastatic lymph nodes.

1.2.2. Patients 2 and 4

YUMC-R-P1 and -P3 were 54- and 34-year-old women with PTC. The patients had multiple tumors and extensive extrathyroidal extension. The patients underwent bilateral total thyroidectomy with central compartment neck dissection. One year after surgery, metastasis to the mediastinum and right lateral cervical lymph nodes was confirmed, and the patient underwent mediastinal dissection through partial sternotomy and right modified radical neck dissection. Specimens for culture were obtained after the final operation. The patients underwent sorafenib treatment, after which disease progression was confirmed in the sorafenib drug response evaluation. Cancer recurrence and metastasis were observed after sorafenib treatment.

1.2.3. Patient 3

YUMC-R-P2 was a 57-year-old man with PDTC. After bilateral total thyroidectomy with central compartment neck dissection, the patient underwent left radical nephrectomy and right lung wedge resection for kidney and lung metastases. Subsequently, he underwent right modified radical neck dissection, left lateral selective lymph node dissection, and two regional lymph node dissections. Specimens for culture were obtained after the final operation. After regional lymph node dissection (left level III), the pathological report indicated the presence of metastatic PDTC. Sorafenib treatment was administered to the patient, after which disease progression was confirmed in the anti-cancer drug response evaluation. Cancer recurrence and metastasis were observed after sorafenib treatment.

1.3. mRNA-Seq data

We preprocessed the raw reads from the sequencer to remove low quality and adapter sequences before analysis and aligned the processed reads to the Homo sapiens genome assembly (GRCh37) using HISAT v2.1.0 (KIM et al, 2015). HISAT utilizes two types of indexes for alignment: a global, whole-genome index, and tens of thousands of small local indexes. Both are constructed using the same Burrows–Wheeler transform (BWT) or graph FM index (GFM) as Bowtie2. Because of the use of these efficient data structures and algorithms, HISAT generates spliced alignments several times faster than Bowtie and the widely used BWA. The reference genome sequence of Homo sapiens (GRCh37) and annotation data were downloaded from the National Center for Biotechnology Information (NCBI). Then, transcript assembly of known transcripts was processed using StringTie v2.1.3b (Pertea, Mihaela, et al., 2015, 2016). Based on these results, expression abundance of transcript and gene were calculated as read count or fragments per kilobase of exon per million fragments mapped (FPKM) value per sample. The expression profiles were used for additional analyses, such as of differentially expressed genes (DEGs). In groups with different conditions, differentially expressed genes or transcripts were filtered through statistical hypothesis testing.

1.4. Statistical analysis of gene expression level

The relative abundances of genes were measured in Read Count using StringTie. We performed statistical analyses to find differentially expressed genes using the estimates of abundances for each gene in the samples. Genes with one more than zeroed Read Count values in the samples were excluded. To facilitate log2 transformation, 1 was added to each Read Count value of filtered genes. Filtered data were log2-transformed and subjected to trimmed mean of M-values (TMM) normalization. The statistical significance of the

differential expression data was determined using exactTest, edgeR and fold change, in which the null hypothesis was that no difference exists among groups. False discovery rate (FDR) was controlled by adjusting the p-value using the Benjamini-Hochberg algorithm. For DEG sets, hierarchical clustering analysis was performed using complete linkage and Euclidean distance as a measure of similarity. Gene-enrichment and functional annotation analysis and pathway analysis for significant gene list were performed based on Gene Ontology and KEGG pathway analyses.

1.5. Intracellular calcium measurements by microspectrofluorimetry

The intracellular Ca^{2+} level of sorafenib-senisitve and -resistant PTC cells were represented with a calcium sensitive fluorescent indicator, Fura-2AM. Cells were incubated with Fura-2AM in normal PBS solution for 40 min at 37 °C, followed by de-esterification of the indicattor for another 40 min at room temperature (22-25°C). Fura-2AM was excited at a wavelength of 340 nm, and emitted light was filtered with a 380 nm band pass filter. Fluorescence intensities (ΔF) were normalized to the resting values. Cells were perfused with 140 mM NaCl, 5.4 mM KCl, 2 mM CaCl2, 1 mM MgCl2, 33 mM glucose, 20 mM HEPES (Ph 7.4, adjusted with NaOH, AND 320-350 Osm with sucrose).

1.6. Human PTC cell xenograft

YUMC-S-P1, YUMC-R-P1, -P2, and -P3 human cells (4.4×10^6 cells/mouse) were cultured *in vitro* and injected subcutaneously into the upper left flank region of female NOD/Shi-scid IL-2Rγ KOJic (NOG) mice. After 13 days, tumor-bearing mice were grouped randomly (n = 10/group) and treated with 25 mg/kg (p.o.) SERCA inhibitors (thapsigargin or

candidate 24 or 31) with 80 mg/kg sorafenib (p.o.) either alone or in combination (excluding the combination of SERCA inhibitors) once every other day (Supplementary Figure 1). Tumor size was measured every other days using calipers. The tumor volume was estimated using the following formula: L × S2/2 (L, longest diameter; S, shortest diameter). Animals were maintained under specific pathogen-free conditions. All the experiments were approved by the Animal Experiment Committee of Yonsei University (2022-0105).

1.7. Statistical analysis

Statistical analyses were performed using GraphPad Prism software (version 6.0; GraphPad Software, La Jolla, CA, USA), Microsoft Excel (Microsoft Corp., Redmond, WA, USA), and R version 2.17. One-way analysis of variance (ANOVA) was performed for multi-group analysis, and a two-tailed Student's *t* test was performed for two-group analysis.

Supplementary Table

Table S1. Primer sequences for qRT-PCR.

Gene	Forward primer (5'-3')	Reverse primer (5'-3')
SERCA1	GTGATCCGCCAGCTAATG	CGAATGTCAGGTCCGTCT
SERCA2	GGTGGTTCATTGCTGCTGAC	TTTCGGACAAGCTGTTGAGG
SERCA3	GATGGAGTGAACGACGCA	CCAGGTATCGGAAGAAGAG
α-tubulin	CGGGCAGTGTTTGTAGACTTGG	CTCCTTGCCAATGGTGTAGTGC

Supplementary Figures

Figure S1. Changes in relative tumor volumes at different doses of so-rafenib or novel SERCA inhibitors (candidate 24 and 31). A; YUMC-S-P1, B and C: YUMC-R-P1. Data are presented as mean ± standard error of mean. * $p < 0.05$ versus control, ** $p < 0.01$ versus control, *; sorafenib, *; C24 and *; C31.

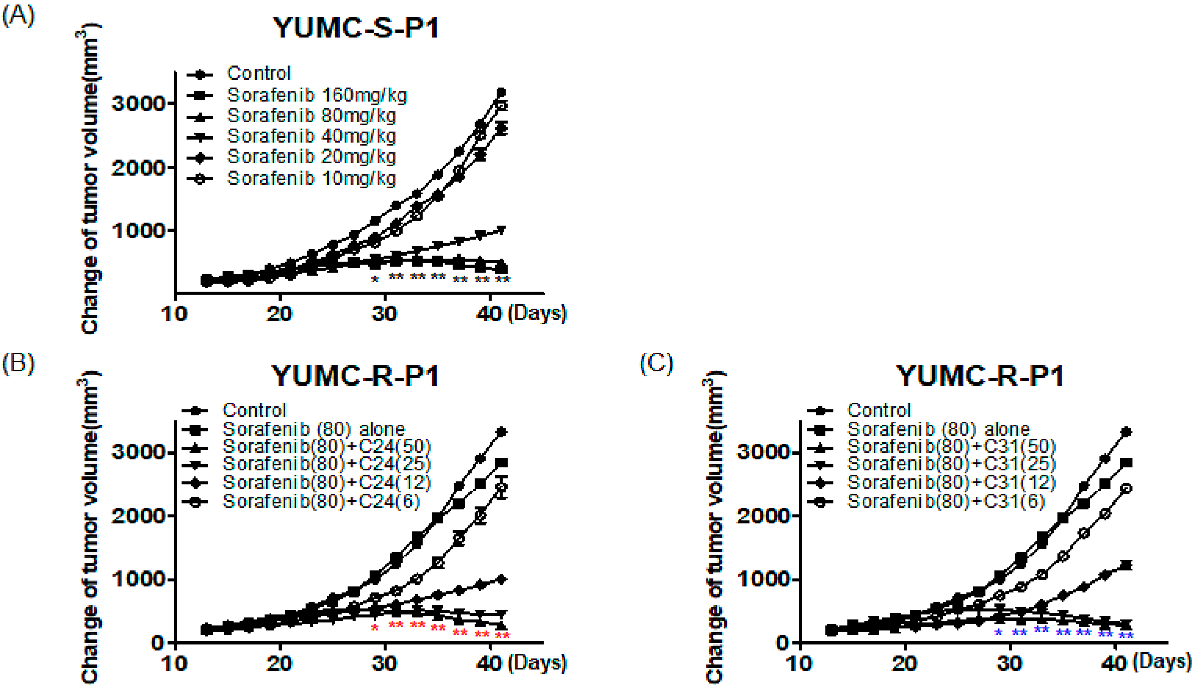

Supplement: Supplementary file 1 [file ijms-24-07069-s001.zip › ijms-2297881-supplementary.pdf]
